# G3BP1-dependent condensation of translationally inactive viral RNAs antagonizes infection

**DOI:** 10.1126/sciadv.adk8152

**Published:** 2024-01-31

**Authors:** James M. Burke, Oshani C. Ratnayake, J. Monty Watkins, Rushika Perera, Roy Parker

**Affiliations:** ^1^ Department of Molecular Medicine, The Herbert Wertheim UF Scripps Institute for Biomedical Innovation & Technology, Jupiter, FL 33458, USA.; ^2^ Department of Immunology and Microbiology, The Herbert Wertheim UF Scripps Institute for Biomedical Innovation & Technology, Jupiter, FL 33458, USA.; ^3^ Center for Vector-Borne and Infectious Diseases, Department of Microbiology, Immunology and Pathology, Colorado State University, Fort Collins, CO 80523, USA.; ^4^ Center for Metabolism of Infectious Diseases, Colorado State University, Fort Collins, CO 80523, USA.; ^5^ Skaggs Graduate School of Chemical and Biological Sciences, The Scripps Research Institute, Jupiter, FL 33438, USA.; ^6^ Howard Hughes Medical Institute, University of Colorado Boulder, Boulder, CO 80303, USA.; ^7^ BioFrontiers Institute, University of Colorado Boulder, Boulder, CO 80303, USA.

## Abstract

G3BP1 is an RNA binding protein that condenses untranslating messenger RNAs into stress granules (SGs). G3BP1 is inactivated by multiple viruses and is thought to antagonize viral replication by SG-enhanced antiviral signaling. Here, we show that neither G3BP1 nor SGs generally alter the activation of innate immune pathways. Instead, we show that the RNAs encoded by West Nile virus, Zika virus, and severe acute respiratory syndrome coronavirus 2 are prone to G3BP1-dependent RNA condensation, which is enhanced by limiting translation initiation and correlates with the disruption of viral replication organelles and viral RNA replication. We show that these viruses counteract condensation of their RNA genomes by inhibiting the RNA condensing function of G3BP proteins, hijacking the RNA decondensing activity of eIF4A, and/or maintaining efficient translation. These findings argue that RNA condensation can function as an intrinsic antiviral mechanism, which explains why many viruses inactivate G3BP proteins and suggests that SGs may have arisen as a vestige of this antiviral mechanism.

## Teaser

G3BP1-dependent condensation of viral RNA is an intrinsic antiviral mechanism in mammalian cells.

## INTRODUCTION

Stress granules (SGs) are condensates composed of untranslating mRNAs and proteins that form when translation initiation is repressed in response to cellular stress (*1*, *2*). Untranslating messenger ribonucleoprotein (mRNP) complexes condense into SGs through a combination of protein-protein, protein-RNA, and RNA-RNA interactions between individual mRNPs (*3*, *4*). Long RNAs are more prone to RNA condensation due to an increased number of possible interaction sites (*5*–*7*).

Key proteins for promoting RNA condensation and SG formation are G3BP1 and its paralog G3BP2 (*8*–*12*). G3BP proteins dimerize through an N-terminal NTF2-like (NTF2L) domain and use RNA binding domains at the C terminus to link RNAs together to form SGs (*8*, *10*–*12*).

G3BP1 and G3BP2 antagonize the replication of numerous and diverse viruses, and many viruses inhibit G3BP proteins (*13*, *14*). For example, the severe acute respiratory syndrome coronavirus 2 (SARS-CoV-2) nucleocapsid (N) protein inhibits G3BP1/2 function by binding the NTF2L domain of G3BP proteins, which then limits SG assembly (*15*–*17*). However, the mechanisms by which G3BP1/2 antagonize viral infection remain unclear.

## RESULTS

### G3BP proteins do not generally alter innate immune responses to double-stranded RNA or viral infection

One model for the antiviral function of G3BP proteins is that they promote the assembly of SGs, which, in turn, enhance recognition of viral RNA [single-stranded RNA or double-stranded RNA (dsRNA)] by concentrating pattern recognition receptors that active innate immune pathways (*13*, *18*–*24*). In addition, G3BP1 also localizes to a distinct RNP complex that forms during the antiviral response, which is termed ribonuclease L (RNase L)–induced body (RLB) (*25*, *26*). Whether RLBs promote innate immune signaling has not been formally addressed.

RLBs can be differentiated from SGs by their smaller and more spherical morphology, as well as their distinct biogenesis pathway. SGs assemble upon protein kinase R (PKR)–mediated repression of translation initiation, which results in untranslating mRNAs condensing into SGs in a G3BP1-dependent manner. In contrast, RLBs form upon the activation of RNase L–mediated mRNA decay and do not require either PKR or G3BP1 for their assembly (*26*). The assembly of SGs and RLBs in cells is generally mutually exclusive because the activation of RNase L, which triggers the assembly of RLBs, inhibits the assembly of SGs via degradation of SG-associated mRNAs (*25*, *26*). Thus, A549 cells only generate RLBs in response to dsRNA because RNase L activation either precedes or coincides with PKR activation, whereas A549 RNase L–knockout (KO) cells only generate SGs in response to dsRNA or viral infection (*25*, *26*).

#### Components of the Rig-I–like–mitochondrial antiviral signaling–interferon regulatory factor 3 pathway do not generally localize to RLBs or SGs

The primary innate immune pathway that G3BP proteins and/or SGs are thought to promote is Rig-I–like–mitochondrial antiviral signaling–interferon regulatory factor 3 (RLR-MAVS-IRF3) pathway. In this pathway, RLR proteins, MDA-5 or RIG-I, bind to viral dsRNA or 5′-ppp RNA, respectively, which leads to activation of the MAVS signaling complex. MAVS signaling results in the phosphorylation of IRF3 (p-IRF3), which translocates to the nucleus to induce immediate-early genes, such as *interferon beta*.

To test whether either SGs or RLBs promote the RLR-MAVS-IRF3 pathway by concentrating its components, we transfected wild-type (WT) or RNase L–KO (RL-KO) A549 cells with polyinosinic:polycytidylic acid [poly(I:C)], a viral dsRNA mimic that triggers MDA-5–mediated activation of the MAVS signaling complex, oligoadenylate synthetase 3 (OAS3)–mediated activation of RNase L, and PKR-mediated SG assembly (*26*–*28*). We then performed immunofluorescence (IF) assays to determine whether the components of the RLR-MAVS-IRF3 pathway colocalize with G3BP1 in RLBs (WT cells) or SGs (RL-KO cells).

**Fig. 1.**
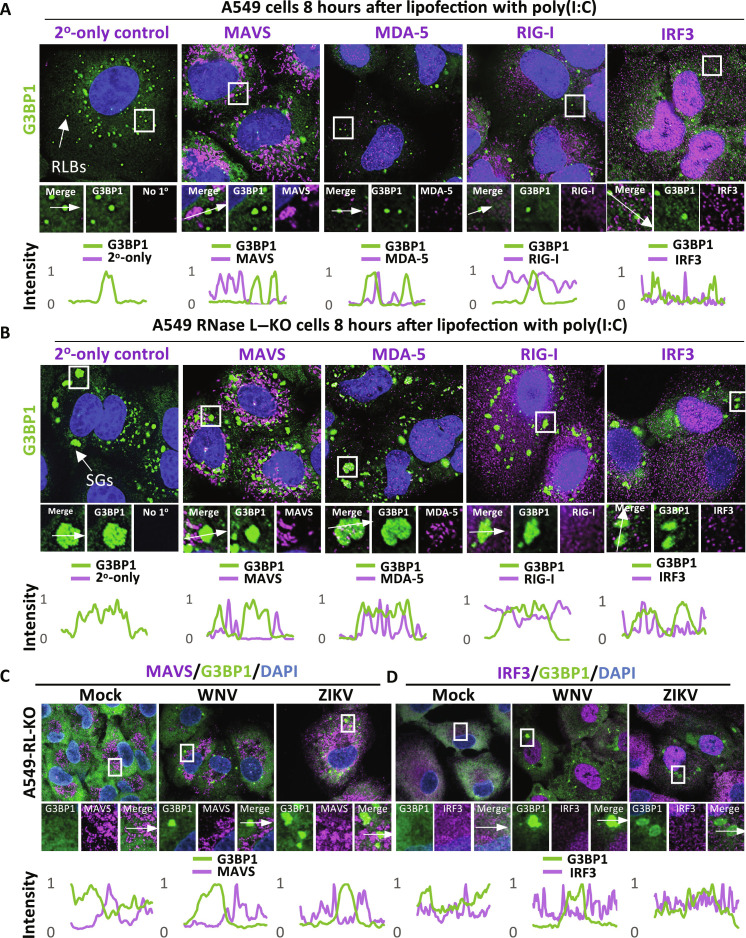
Components of the RLR-MAVS-IRF3 pathway do not enrich in G3BP1 complexes. (**A**) IF assays for G3BP1, Rig-I, MDA-5, MAVS, and IRF3 in A549 cells 8 hours after poly(I:C) lipofection. (**B**) Similar to (A) but in A549 RL-KO cells. (**C**) IF assays for G3BP1 and MAVS in RL-KO A549 cells 24 hours after infection with either WNV or ZIKV [multiplicity of infection (MOI) = 10]. (**D**) IF assays for G3BP1 and IRF3 in RL-KO A549 cells 24 hours after infection with either WNV or ZIKV (MOI = 10). The graphs below image panels represent intensity values (*y* axis) across the line profile (*x* axis) shown in the merged inset. Intensity values were normalized to the maximum value across the line profile.

RIG-I, IRF3, MDA-5, or MAVS did not colocalize with G3BP1 in RLBs in WT cells 8 hours after lipofection of poly(I:C) (Fig. 1A). Similarly, RIG-I, MAVS, or IRF3 did not localize to SGs in RL-KO cells (Fig. 1B). We did observe slight enrichment of MDA-5 in SGs (Fig. 1B), consistent with a previous report (*29*). We confirmed specificity of our MAVS staining via a second MAVS-specific antibody (fig. S1A), and CRISPR-mediated KO of MAVS (fig. S1B). We also observed nuclear signal for IRF3 (Fig. 1, A and B), which is consistent with its relocalization to the nucleus upon activation of MAVS signaling (see below) and indicates the IF signal was specific to IRF3.

We also tested whether MAVS and IRF3 localize to SGs in response to viral infection. However, we did not observe enrichment of MAVS or IRF3 with SGs assembled in response to Zika virus (ZIKV) or West Nile virus (WNV) infection (Fig. 1, C and D). Combined, these data indicate that components of the RLR-MAVS-IRF3 pathway do not highly enrich in G3BP1 RNP complexes (SGs/RLBs) during the innate immune response to dsRNA or viral infection.

#### G3BP1 proteins do not alter RLR-MAVS-IRF3 signaling

**Fig. 2.**
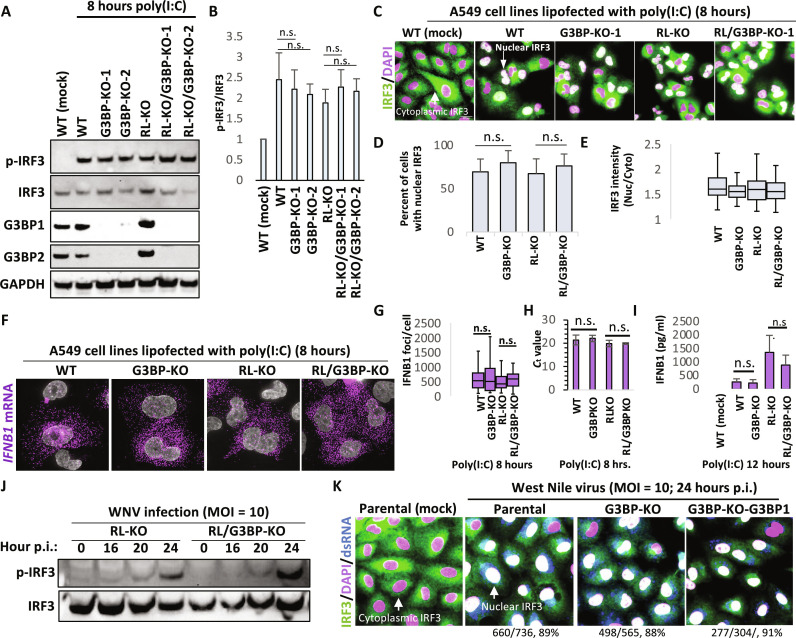
G3BP proteins do not alter activation of the RLR-MAVS-IRF3 pathway. (**A**) Western blot (WB) for indicated proteins. G3BP1 and G3BP2 paralogs were knocked out in parental (WT) cells or RL-KO A549 cells. Two clonal G3BP-KO cell lines were analyzed for p-IRF3 in response to poly(I:C) lipofection (8 hours). (**B**) Graph displaying the average p-IRF3:IRF3 band intensity ± SD from three independent experiments as represented in (A). (**C**) IF for IRF3 8 hours after poly(I:C) lipofection. (**D**) Graph represents the average percentage of cells displaying nuclear IRF3 signal per field of view as represented in (C). Greater than 100 cells were analyzed from at least 10 fields of view. (**E**) Intensity of IRF3 nuclear staining as represented in (C). (**F**) Single-molecule fluorescence in situ hybridization (smFISH) for *interferon beta* mRNA (*IFNB1*) 8 hours after lipofection of poly(I:C) in indicated cell lines. Clone 1 for G3BP-KO and RL/G3BP-KO was used. (**G**) Quantification of IFNB1 mRNA as represented in (E). (**H**) Average quantitative reverse transcription polymerase chain reaction (qRT-PCR) *C*_t_ value ± SD from three independent for *IFNB1* mRNA 8 hours after lipofection of poly(I:C). Clone 1 for G3BP-KO and RL/G3BP-KO was used. (**I**) Enzyme-linked immunosorbent assay (ELISA) for IFNB protein secretion 12 hours after lipofection of poly(I:C). Clone 1 for G3BP-KO and RL/G3BP-KO was used. Bars represent the average ± SE of five independent replicates. (**J**) WB for p-IRF3 at indicated times after infection (p.i.) of RL-KO (parental) or RL/G3BP-KO (G3BP-KO) A549 cells with WNV (MOI = 10). (**K**) IF assay for IRF3 in A549 cells 24 hours after infection with WNV (MOI = 10). Clone 1 for G3BP-KO and RL/G3BP-KO was used. G3BP1 expression was rescued in G3BP-KO cells viral lentiviral transduction (G3BP-KO-Rescue). Displayed below the panels are the number of cells displaying nuclear IRF3, the total number of cells analyzed, and the percentage of cells displaying nuclear IRF3. n.s., not significant.

We next tested whether G3BP proteins alter RLR-MAVS-IRF3 signaling. We knocked out G3BP1/2 proteins in WT (G3BP-KO) and RL-KO (RL/G3BP-KO) A549 cell lines (Fig. 2A), which abolished poly(I:C)- and sodium arsenite–induced SG assembly (fig. S2, A and B). We then examined IRF3 activation (phosphorylation and nuclear translocation) and interferon beta (IFNB) mRNA induction 8 hours after lipofection of poly(I:C), which is when the peak number of A549 cells that initiate RLR-MAVS-IRF3 signaling occurs (*25*, *30*).

In response to poly(I:C), KO of G3BP proteins in either WT or RL-KO cells did not alter p-IRF3 levels (Fig. 2, A and B, and fig. S3, A and B), nuclear localization of IRF3 (Fig. 2, C to E), the induction of IFNB mRNA (Fig. 2, F to H), or secretion of IFNB protein (Fig. 2I). G3BP expression also did not alter p-IRF3 levels in response to 5′-tri-phosphorylated T7-generated dsRNA (fig. S3C), which is primarily recognized by RIG-I to initiate MAVS signaling (*27*). The rescue of G3BP1 expression in the G3BP-KO cells also did not alter p-IRF3 levels (fig. S3, B and C).

We also tested whether G3BP1 proteins altered RLR-MAVS-IRF3 signaling in response to viral infection. We observed no difference between parental and G3BP-KO A549 cells in the kinetics of p-IRF3 in response to WNV infection (Fig. 2J). Consistent with this observation, G3BP1 expression had no effect on the percentage cells displaying nuclear IRF3 in response to WNV (Fig. 2K) or ZIKV infection (fig. S3D).

Because RLB assembly is not abolished in G3BP-KO A549 cells (fig. S2B) (*26*), we tested whether RLBs promote RLR-MAVS-IRF3 signaling by examining A549 cells lacking both PKR and RNase L (RL/PKR-KO), which do not assemble either RLBs or SGs (*26*). In comparison to cells that assemble RLBs (WT and PKR-KO cells) or SGs (RL-KO cells), RL/PKR-KO (RLB/SG-null cells) did not display a reduction in nuclear IRF3 (fig. S4, A and B), induction of IFNB (fig. S4A), or secretion of IFNB protein (fig. S4C). These data argue that neither G3BP proteins nor G3BP complexes (SGs or RLBs) are required for RLR-MAVS-IRF3 signaling.

We also attempted to examine p-IRF3 in response to poly(I:C) in parental (WT) and G3BP1/2-KO U-2 OS cells, which are commonly used to study SG biogenesis and function (*9*). However, transfection of poly(I:C) in parental or G3BP-null U-2 OS cell lines resulted in very low or undetectable p-IRF3 (fig. S5, A to C) and secretion of IFNB protein (fig. S5D). Consistent with this, we could only detect nuclear IRF3 staining in 5% of U-2 OS cells following poly(I:C) lipofection via IF assays (fig. S5E). This result was not due to a failure in transfection of poly(I:C) because we observed robust activation of RNase L based on the assembly of RLBs (fig. S5E). Immunoblot analyses revealed that U-2 OS cells express low levels of RIG-I, IRF3, and MAVS in comparison to A549 cells, which likely accounts for the lack of RLR-MAVS signaling (fig. S5, A to C). Nevertheless, we observed that G3BP1/2-KO U-2 OS cells displayed equivalent nuclear IRF3 staining to parental U-2 OS cells (fig. S5E). Together, these data indicate that G3BP proteins do no alter RLR-MAVS-IRF3 signaling for type I interferon production in response to dsRNA or flavivirus infection.

#### G3BP proteins do not alter RNase L or PKR activation

**Fig. 3.**
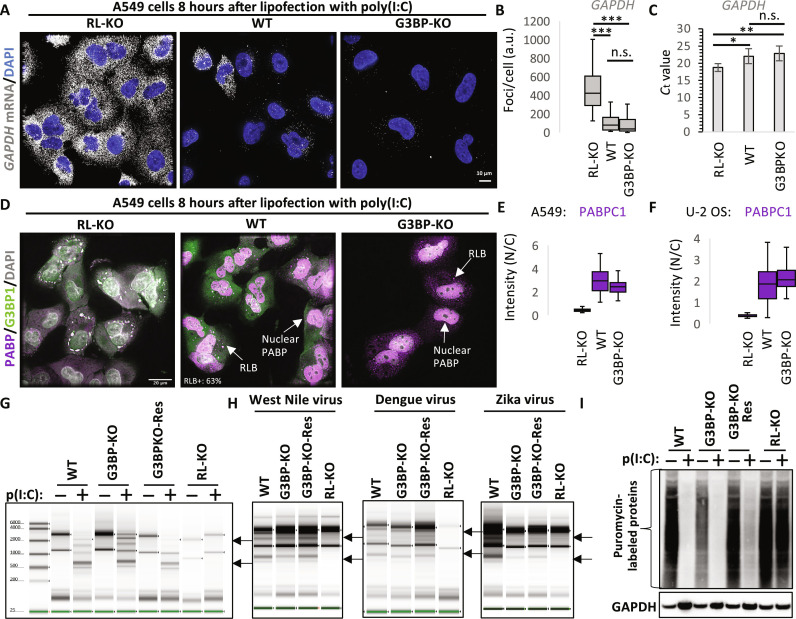
G3BP proteins do not alter activation of RNase L. (**A**) smFISH for *GAPDH* mRNA 8 hours after lipofection of poly(I:C) in parental (WT), G3BP-KO, or RL-KO A549 cell lines. (**B**) Quantification of *GAPDH* mRNA smFISH as represented in (A) from 44 WT cells and 73 RL-KO cells from at least four fields of view. (**C**) Average qRT-PCR *C*_t_ value ± SD from three independent for *GAPDH* mRNA 8 hours after lipofection of poly(I:C). (**D**) IF for PABPC1 and G3BP1 in indicated A549 cell lines. (**E**) Quantification of the intensity of PABPC1 staining in the nucleus in A549 cells as represented in (D) from three fields of view. (**F**) Similar to (D), except U-2 OS cells were analyzed. (**G**) RNA TapeStation analysis of ribosomal RNA in indicated cell lines following poly(I:C) lipofection. Arrows indicate RNase L–dependent ribosomal RNA cleavage fragments. (**H**) Similar to (G) but 24 hours after infection with WNV, ZIKV, or dengue virus serotype 2 (MOI = 1). (**I**) Puromycin labeling of nascent peptides 6 hours after lipofection of poly(I:C). a.u., arbitrary units. *P* values were derived via Student’s *t* test. *P < 0.05, **P < 0.01, and ***P < 0.001.

We also observed no difference between WT and G3BP-KO A549 cells for the activation of RNase L as assessed by single-molecule fluorescence in situ hybridization (smFISH) or reverse transcription quantitative polymerase chain reaction (qRT-PCR) for *GAPDH* mRNA (Fig. 3, A to C), Poly(A) Binding Protein Cytoplasmic 1 (PABPC1) translocation to the nucleus (Fig. 3, D to F), assembly of RLBs (Fig. 3D), cleavage of ribosomal RNAs in response to poly(I:C) or dengue virus, ZIKV, or WNV infection (Fig. 3, G and H), or translation repression (Fig. 3I). These observations are consistent with the fact that RNase L activation precedes both RLB and SG assembly in response to dsRNA (*25*, *26*) and that neither OAS3 or RNase L localizes to SGs or RLBs (*31*).

Similarly, we observed that G3BP proteins are not required for the activation of PKR, which is activated via autophosphorylation upon binding dsRNA and limits translation by phosphorylating eukaryotic translation initiation factor 2 subunit alpha (eIF2α) (*32*, *33*). G3BP-KO cells did not display differences in either p-PKR or p-eIF2α in response to poly(I:C) (fig. S6, A to C), T7-generated 5′-ppp-dsRNA (fig. S6B), or infection with either WNV or ZIKV (fig. S6D). These observations are consistent with studies showing that PKR activation precedes SG assembly and that PKR does not localize to SGs or RLBs (*26*, *34*, *35*).

Combined, our above data argue that neither G3BP proteins nor G3BP1 complexes (SGs or RLBs) alter the activation of the RLR-MAVS-IRF3, OAS/RNase L, or PKR innate immune pathways in response to dsRNA lipofection or flavivirus infection, which contrasts with previous studies (*18*–*24*, *36*). While these data argue that G3BP proteins are generally not involved in regulating these innate immune signaling pathways, we acknowledge the possibility that G3BP proteins could regulate these innate immune pathways in response to different viruses (i.e., influenza A virus), in specific cell types, or could regulate other innate immune antiviral pathways. Future studies will more broadly address these possibilities. Nevertheless, these data led us to test alternative antiviral functions of G3BP1.

### G3BP1 promotes condensation of translationally repressed viral RNAs

We hypothesized that G3BP proteins exert an antiviral effect by condensing viral RNAs based on three aspects of viral infections. First, viral RNAs are generally long and thus prone to RNA condensation (*5*, *6*). For example, the SARS-CoV-2 RNA genome (~30 kb) is ~14-fold longer than the average cellular mRNA (2.2 kb). Second, viral RNAs are abundant, which promotes RNA condensation. During SARS-CoV-2 infection, cells contain 100,000 to 1 million copies of the genomic RNA (*37*). Because mammalian cells contain around ~330,000 mRNAs with an average length of 2.2 kb (*5*), this leads to ~3 to 30 times higher concentrations of mRNAs by mass than typically occurs in a mammalian cell, even without considering subgenomic viral RNAs. Third, host cells try to limit translation during viral infection, which is expected to increase RNA condensation by increasing the pool of nontranslating host and/or viral mRNPs. This hypothesis predicts that (i) preventing viral inhibition of G3BP function would increase viral RNA condensation, (ii) decreasing translation during viral infections would enhance viral RNA condensation, and (iii) decreasing the helicase function of the DEAD-box protein, eukaryotic initiation factor-4A (eIF4A), which limits the condensation of RNA by destabilizing short RNA duplexes (*38*), would also enhance viral RNA condensation.

#### Reducing SARS-CoV-2 N binding to G3BP1 promotes G3BP1-mediated viral RNA condensation

**Fig. 4.**
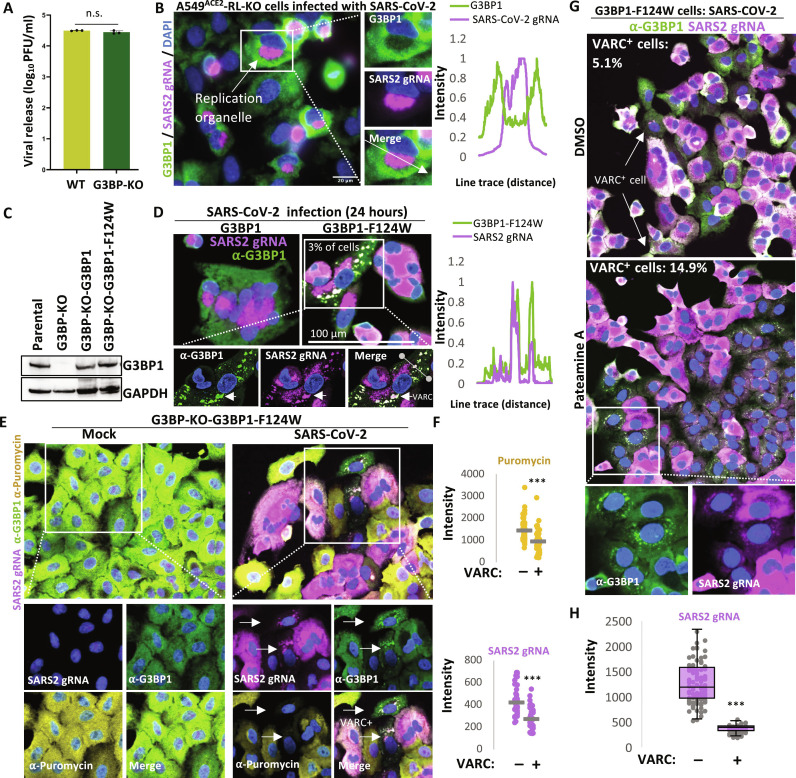
G3BP1-F124W promotes SARS-CoV-2 RNA condensation when translation is reduced. (**A**) Plaque assay for SARS-CoV-2 release from A549^ACE2^-RL-KO parental or G3BP-KO cell lines 48 hours after infection (MOI = 5). (**B**) IF assay for G3BP1 and smFISH for SARS-CoV-2 genomic RNA (gRNA) in cells 24 hours after infection with SARS-CoV-2. (**C**) WB showing G3BP-1 in A549^ACE2^-RL-KO (parental), G3BP1/2-KO cells, or G3BP-KO cells transduced with lentivirus encoding G3BP1 or G3BP1-F124W. (**D**) IF assay for G3BP1 in G3BP-KO cells expressing G3BP1 or G3BP1-F124W 24 hours after infection. Line profile plot is displayed on the right. (**E**) Ribopuromycylation assay in which IF for G3BP1 and puromycin and smFISH for SARS-CoV-2 ORF1a RNA was performed in G3BP-KO cells expressing G3BP1-F124W 24 hours after infection. Arrows indicate cells that are VARC-positive and display low translation. (**F**) Quantification of puromycin or SARS-CoV-2 gRNA in SARS-CoV-2–infected cells (positive for viral RNA) that did (+) or did not (−) contain VARCs. Eighty-seven cells were analyzed, each represented by a dot. Gray bars represent the average intensity. (**G**) IF for G3BP1 and smFISH for SARS-CoV-2 ORF1a in cells infected for 20 hours and then treated with 10 nM pateamine A for 4 hours. Cells were identified as VARC^+^ if they contained G3BP1 granules that costained for viral RNA above background. Three fields of view were analyzed, and 724 and 447 G3BP1 and G3BP1-F1234W cells were analyzed in total. DMSO, dimethyl sulfoxide. (**H**) Quantification of the mean intensity of SARS-CoV-2 RNA in VARC^+^ or VARC^−^ cells; 153 cells were analyzed.* P* values were derived via Student’s *t* test. *P < 0.05, **P < 0.01, and ***P < 0.001.

The SARS-CoV-2 N protein binds G3BP1 (*15*–*17*, *39*). Therefore, we considered the possibility that G3BP1 is a host factor that promotes SARS-CoV-2 infection, as occurs during norovirus infection (*40*). However, we observed equivalent plaque forming units from parental and G3BP-KO cells (Fig. 4A), indicating that G3BP is not required for SARS-CoV-2 replication.

We hypothesized that SARS-CoV-2 N protein inhibits G3BP1 to limit viral RNA condensation. In support of this hypothesis, we observed that G3BP1 does not assemble into SG structures during SARS-CoV-2 infection (Fig. 4B). Moreover, G3BP1 was excluded from the replication organelle (RO) that enriches for SARS-CoV-2 genomic RNA (Fig. 4B). The lack of SG assembly observed in SARS-CoV-2–infected cells was not due to a lack of p-eIF2α, which promotes SG assembly, based on two observations. First, IF assay for p-eIF2α revealed that nearly every SARS-CoV-2–infected cell induces p-eIF2α and that the level of p-eIF2α positively correlated with the level of viral RNA (fig. S7A). Second, treatment of SARS-CoV-2–infected cells with sodium arsenite, which induces p-eIF2α and SG assembly, did not result in SG assembly in SARS-CoV-2–infected cells (fig. S7B). Combined, these data indicate that SARS-CoV-2 N protein inhibits G3BP1, resulting in an inhibition of RNA condensation, which is consistent with earlier reports of N protein inhibiting SG formation (*15*–*17*, *39*).

We tested whether decreasing the ability of SARS-CoV-2 N protein to inhibit G3BP proteins could promote G3BP1-mediated condensation of SARS-CoV-2 RNA. The G3BP1-F124W mutant is functional in promoting SGs yet resistant to binding SARS-CoV-2 N protein (*41*). Therefore, we stably expressed at normal levels both WT and the N-resistant G3BP1 protein (G3BP1-F124W) in A549^ACE2^-RL/G3BP-KO cells (Fig. 4C).

We observed that ~3% of infected cells expressing G3BP1-F124W formed large RNP granules containing G3BP1-F124W and SARS-CoV-2 genomic RNA (Fig. 4D). Because host mRNAs are degraded at this time of infection (*42*) and these RNP granules contain viral RNAs, we term them viral aggregated RNA condensates (VARCs). VARCs are defined as irregularly shaped assemblies that contain viral gRNA and G3BP1 and are not seen during SARS-CoV-2 infections in cells expressing the WT G3BP1 protein (Fig. 4D). These data argue that reducing SARS-CoV-2 N protein interactions with G3BP1 results in an increase in G3BP1 interactions with SARS-CoV-2 gRNA, which leads to viral RNA condensation.

#### G3BP1 promotes viral RNA condensation upon reduced translational efficiency

The observation that only some cells expressing G3BP1-F124W produced VARCs during infections suggested individual cell variations can affect condensation of the viral gRNAs. One variable is the translation capacity of cells, which can widely vary because of host factors such as Interferon Induced Protein With Tetratricopeptide Repeats (IFIT) proteins, Interferon-stimulated gene 20 (ISG20), PKR, and RNase L (*25*, *43*, *44*). Ribopuromycylation assays revealed marked differences in translation activity between individual cells infected with SARS-CoV-2 (Fig. 4E and fig. S7A). In contrast, mock-infected cells showed relatively uniform and high translation (Fig. 4E and fig. S7A). Because we performed this assay after host mRNAs have been degraded (*42*), this argues that individual cells show high variability in the translation of viral mRNAs.

We observed that VARC assembly in individual cells correlated with lower translation rate as assessed by puromycin staining (Fig. 4, E and F). These data are consistent with the hypothesis that reduced translation would enhance viral RNA condensation. Moreover, cells with VARCs had less gRNA on average, suggesting that VARC inhibits viral RNA production (Fig. 4, E and F).

To directly test whether reducing translation would promote VARC formation, we reduced translation initiation with low doses (10 nM) of the eIF4A inhibitor pateamine A, which inhibits eIF4A’s role in translation initiation but allows eIF4A to be active as an RNA helicase and thereby decondense RNA (*38*). We observed that ~15% of G3BP-KO cells expressing G3BP1-F124W and treated with pateamine A displayed VARCs (Fig. 4G), which is fivefold increase from nontreated cells (Fig. 4D). Viral RNA levels in cells containing VARCs were threefold lower in comparison to cells that did not contain VARCs (Fig. 4H), indicating that VARC formation limits viral RNA production. This RNA condensation is limited by the viral N protein since in cells expressing WT G3BP1, which is inhibited by the N protein, we observed that ~5% of the cells formed smaller VARCs. These data indicate that translation of viral RNA limits RNA condensation even in the presence of the viral N protein (Fig. 4G), potentially by disrupting G3BP1-RNA and/or RNA-RNA interactions that drive G3BP1-mediated RNA condensation.

#### Inhibition of eIF4A RNA helicase activity promotes condensation of SARS-CoV-2 RNA

The helicase activity of eIF4A inhibits RNA condensation and SG assembly independently of its role in promoting translation (*38*). Thus, we tested whether inhibition of eIF4A helicase activity promoted VARC formation mediated by endogenous G3BP1 by treatment with hippuristanol, which blocks eIF4A function in both translation initiation and in limiting RNA condensation (*38*). We tested this early during infection (8 hours after infection) when N protein levels are expected to be lower relative to the viral genomic RNA. To differentiate the helicase activity and translation initiation functions of eIF4A, we also treated cells with pateamine A, which inhibits eIF4A-mediated translation initiation, but not the helicase and RNA decondensing activity of eIF4A (*38*).

**Fig. 5.**
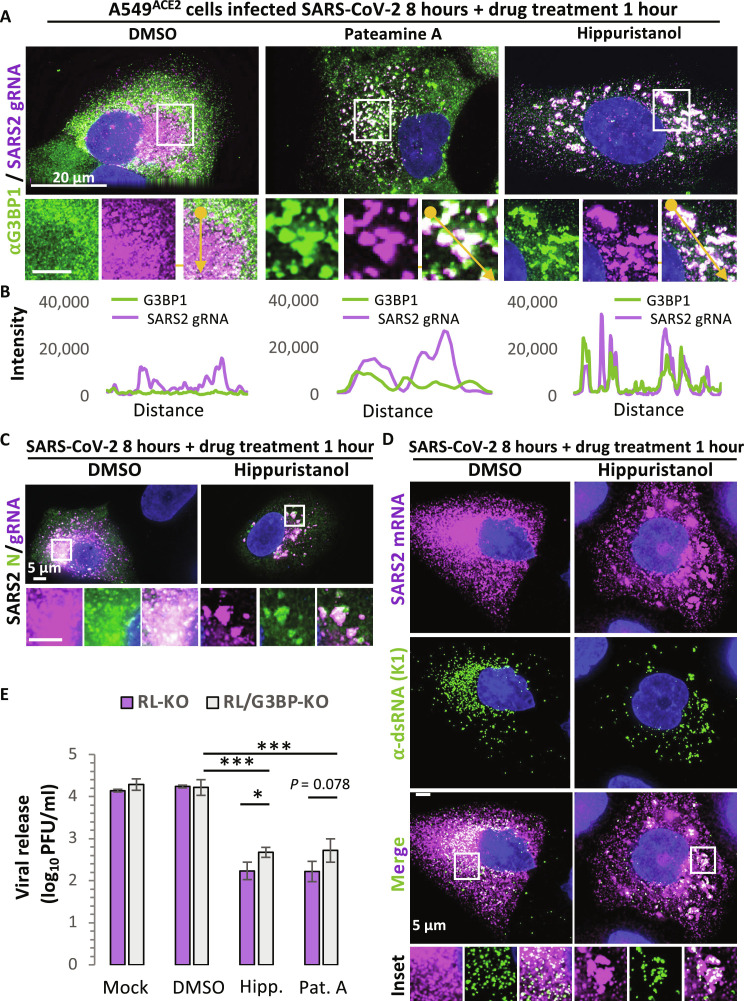
Inhibition of eIF4A promotes condensation of G3BP1 and SARS-CoV-2 RNA. (**A**) smFISH for SARS-CoV-2 ORF1a RNA and IF for G3BP1 in A549^ACE2^ cells infected with SARS-CoV-2 for 8 hours and then treated with either vehicle (DMSO), hippuristanol, or pateamine A for 1 hour. (**B**) Line traces (yellow arrow in merged inset) of fluorescence intensity from images in (A). (**C**) IF for SARS-CoV-2 N protein and smFISH for SARS-CoV-2 ORF1a mRNA/genome. (**D**) IF for SARS-dsRNA via the K1 antibody and smFISH for SARS-CoV-2 ORF1a mRNA/genome. (D). (**E**) Quantification of SARS-CoV-2 viral release from either parental or G3BP-KO via plaque assay after 24 hours of treatment with DMSO (vehicle), hippuristanol, or pateamine A. Bars represent the average ± SD of three independent experiments. *P* values were derived via Student’s *t* test. *P < 0.05, **P < 0.01, and ***P < 0.001.

We did not observe G3BP1 granules consistent with VARCs in vehicle-treated SARS-CoV-2–infected cells (Fig. 5, A and B, and fig. S8). Instead, SARS-CoV-2 gRNA was both widely dispersed in the cytosol and/or concentrated in a large perinuclear RO, as defined by high concentration of both genomic viral RNA and dsRNA (see below) (Fig. 5A). Inhibition of translation initiation with pateamine A led to the formation of small VARCs in infected cells that contained the viral RNA and G3BP1 (Fig. 5, A and B), which is consistent with limiting translation leading to cocondensation of G3BP1 and SARS-CoV gRNA.

Notably, we observed that hippuristanol treatment resulted in numerous large G3BP1 granules in SARS-CoV-2–infected cells that strongly stained for SARS-CoV-2 gRNA (Fig. 5, A and B, and fig. S8). Because hippuristanol and pateamine A both inhibit translation to similar degrees (*38*) and we observed more robust gRNA condensation with hippuristanol treatment, this argues eIF4A also directly limits SARS RNA condensation.

We observed the SARS-CoV-2 N protein relocalized from the viral RO to VARCs (Fig. 5C), demonstrating that VARCs can contain viral proteins. Moreover, we observed viral dsRNA, which is typically localized in perinuclear viral RO, localized in VARCs dispersed throughout the cytoplasm (Fig. 5D). These data demonstrate that inhibition of eIF4A via hippuristanol treatment alters the morphology and localization of viral replication factory components, including viral replication dsRNA intermediates.

#### VARC assembly correlates with G3BP1-dependent reduced SARS-CoV-2 output

The localization of G3BP1 and the SARS-CoV-2 RNA into VARCs led us to test whether G3BP proteins interfere with viral production. We measured viral output in parental and G3BP-KO cells with or without treatment with hippuristanol or pateamine A. Viral output was fivefold higher in G3BP-null cells in comparison to the parental cells treated with hippuristanol or pateamine A (Fig. 5E). These data indicate that G3BP enhances the antiviral effect of pateamine A– or hippuristanol-mediated inhibition of eIF4A.

Notably, both pateamine A and hippuristanol increased G3BP1 colocalization with SARS-CoV-2 RNA (Fig. 5, A and B). This latter observation argues that ongoing translation initiation helps to limit G3BP interaction with SARS-CoV-2 RNAs, as observed for host mRNAs (*10*), consistent with our results with pateamine A and G3BP1-F124W (Fig. 4G).

These observations argue that G3BP can promote RNA condensation of SARS-CoV-2 gRNA to a greater extent when translation is repressed and that eIF4A can also function directly as an RNA helicase to limit SARS RNA condensation. This condensation of viral RNA correlates with a reduction of the viral output arguing that RNA condensation of viral RNAs is inhibitory for viral production. This suggests an ongoing host-pathogen arms race wherein infected cells promote viral RNA condensation by reducing translation and expressing G3BP proteins, while viruses can counter RNA condensation mechanisms by inhibiting G3BP, using RNA decondensors such as eIF4A, and escaping host translation inhibitory mechanisms. One anticipates that diverse mechanisms will be used by viruses to either promote efficient translation or directly combat RNA condensation including inhibiting other RNA condensors such as caprin-1, which is inhibited by Japanese encephalitis virus (*45*), and the use of both viral encoded and host RNA decondensors to limit viral RNA condensation.

#### G3BP1 promotes condensation of translationally repressed flavivirus mRNAs

We hypothesized that many viral RNAs would be prone to G3BP-mediated RNA condensation but escape by evading host translation repression mechanisms. A primary mechanism by which cells limit bulk translation is via the integrated stress response, during which eIF2α kinases [PKR, PRKR-Like Endoplasmic Reticulum Kinase (PERK), GCN2, and Heme-Regulated Inhibitor (HRI)] phosphorylate eIF2α at serine-51 (p-eIF2α). The p-eIF2α limits canonical cap-dependent translation of bulk cellular mRNAs, which leads to condensation of long, nontranslating mRNAs into SGs (*5*). However, p-eIF2α also promotes noncanonical translation of stress response mRNAs, and many viruses have evolved translation initiation mechanisms to escape host-mediated translation repression. Thus, we hypothesized that viral mRNAs/genomes avoid RNA condensation by escaping p-eIF2α–mediated translational repression. If this were the case, then eIF2α-independent repression of translation would lead to G3BP1-mediated viral RNA condensation.

**Fig. 6.**
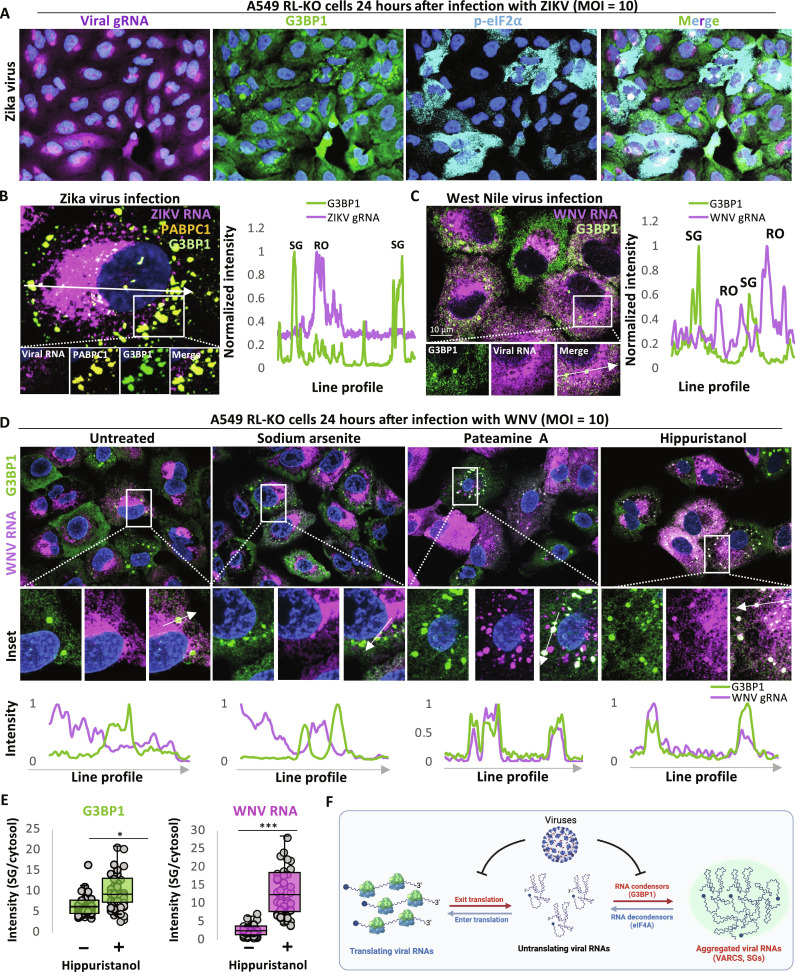
Inhibition of flavivirus mRNA translation results in G3BP1-dependent viral RNA condensation. (**A**) IF for G3BP1 and p-eIF2α and smFISH for the 5′-end of viral RNAs in A549 RL-KO cells 24 hours after infection with ZIKV (MOI = 10). (**B**) High-resolution imaging of IF for G3BP1, PABPC1, and ZIKV gRNA. The line plot profile is indicated by the arrow in the image panel. (**C**) Similar to (B) but for WNV. (**D**) IF for G3BP1 and smFISH for WNV RNA in A549 RL-KO cells. Twenty-three hours after infection, cells were treated with 500 μM sodium arsenite, 50 nM pateamine A, or 1 μM hippuristanol for 1 hour. Plot profiles below are indicated by the line in the merged inset panels. (**E**) Quantification of the intensity of G3BP1 and WNV RNA in SGs or the cytosol with or without hippuristanol treatment as represented in (D). Forty cells from six fields of view were analyzed. *P* values were derived via Student’s *t* test. *P < 0.05, **P < 0.01, and ***P < 0.001. (**F**) Model for factors that regulate viral RNA condensation. Viral RNAs are long and highly concentrate in cells and thus are prone to RNA condensation mediated by G3BP1 proteins. However, viral-mediated inhibition of G3BP1 proteins, efficient translation of viral RNAs, and the RNA decondensor function of eIF4A prevent the condensation of viral RNA.

Given this, we examined p-eIF2α and viral RNA condensation in A549 cells infected with the WNV or ZIKV. To detect full-length viral genomes, we performed smFISH for the 5′-end of viral genomic RNA. At 24 hours after infection, we observed that 40% of WNV-infected cells (fig. S9) and 10% of ZIKV-infected cells contained SGs (Fig. 6A). Nearly all infected cells containing SGs stained positive for p-eIF2α (Fig. 6A and fig. S9), indicating that these cells have activated the host translation shut-off mechanism. Both ZIKV and WNV RNAs were depleted from SGs (Fig. 6, B and C). Moreover, G3BP1 did not enrich in viral ROs (Fig. 6, B and C). These observations suggest that interactions between flavivirus gRNAs and G3BP1 are limited during p-eIF2α–mediated translational arrest. We confirmed that ZIKV-induced SGs are canonical SGs that enriched PABPC1 (Fig. 6B), which indicates that these SGs contain polyadenylated RNAs and thus are composed of nontranslating cellular mRNAs because flavivirus mRNAs lack polyadenylate tails (*46*). These observations imply that both WNV and ZIKV gRNAs escape translation repression by p-eIF2α and their continued translation prevents their condensation into SGs.

If viral translation escape of p-eIF2α is what limits WNV and ZIKV RNA condensation into SG, then we predicted that inhibiting translation by other mechanisms should lead to condensation of these viral RNAs. Thus, we treated infected cells with the eIF4A inhibitors, pateamine A, or hippuristanol and compared that to sodium arsenite, which further increases p-eIF2α levels. Consistent with viral RNAs being resistant to p-eIF2α–mediated translational repression, we observed that cells treated with sodium arsenite showed normal distributions of WNV or ZIKV RNAs in ROs. Moreover, viral RNA remained excluded from SGs with sodium arsenite treatment (Fig. 6D and fig. S10A). We note that sodium arsenite treatment increased the levels of p-eIF2α and the number of ZIKV-infected cells containing SGs (fig. S10B).

Treatment with pateamine A led to increased RNA-protein condensation in cells with some SGs now recruiting the viral gRNA (Fig. 6D and fig. S10A), arguing that translation is partially limiting the condensation of WNV and ZIKV RNAs. Most notably, treatment with hippuristanol led to the formation of G3BP1 assemblies in most cells that costained for viral RNA (Fig. 6D). Hippuristanol treatment resulted in a small (less than twofold), yet significant, increase in G3BP1 partitioning in SGs (Fig. 6E). However, hippuristanol treatment resulted in a 12-fold enrichment of viral RNA in SGs in comparison to the cytoplasm (Fig. 6E), which was significantly higher than nontreatment. The condensation of viral RNA in SGs upon hippuristanol treatment is dependent on G3BP1, as we did not observe viral RNA aggregates that colocalize with Ubiquitin Associated Protein 2 Like (UBAP2L) (SG marker) in G3BP-KO cells (fig. S11), and rescue of G3BP1 expression in G3BP-KO cells rescued viral RNA aggregation in SGs (fig. S11). Notably, we did not observe marked incorporation of dsRNA in SGs with or without hippuristanol treatment, although we did observe large dsRNA aggregates adjacent to SGs (fig. S12). The increased viral RNA condensation seen with hippuristanol argues that the RNA helicase activity of eIF4A limits WNV and ZIKV RNA condensation.

## DISCUSSION

Our data demonstrate that the full-length genomic RNAs encoded by SARS-CoV-2, ZIKV, and WNV are prone to G3BP1-mediated RNA aggregation (Fig. 6F). These findings are consistent with the large size and high abundance of viral RNAs during infection, which are known to promote G3BP1-mediated RNA condensation (*5*, *6*). We term these viral RNA-G3BP1 condensates VARCs.

Several observations argue that viral RNA condensation into VARCs is inhibitory for viral replication. First, we observed reduced levels of SARS-CoV-2 viral RNA in VARC-positive cells expressing F124W-G3BP, correlating viral RNA condensation with reduced viral production (Fig. 4, E and H). Second, we observed that the decrease in SARS-CoV-2 viral production when translation was reduced was partially dependent on G3BP and correlated with viral RNA condensation (Fig. 5). Moreover, an SARS-CoV-2 virus expressing a N-F17A mutant that cannot bind and inhibit G3BP proteins leads to decreased viral production in hamsters and leads to condensation of the SARS-CoV-2 gRNA with G3BP in cell lines (*40*).

Because viral RNAs are inherently prone to RNA condensation, it would be expected that viruses have evolved to counteract RNA condensation. Consistent with this notion, we observed that SARS-CoV-2, WNV, and ZIKV counteract G3BP1-mediated aggregation of their RNAs by maintaining efficient translation despite host stress responses that lead to p-eIF2α (Figs. 5A and 6A and fig. S10), inhibiting G3BP proteins (Fig. 4D), and/or hijacking the RNA decondensing function of eIF4A (Figs. 5A and 6D).

We propose that cells can use RNA condensation as an antiviral defense and that G3BP proteins are key components of this unique innate immune defense system. Because cell-mediated condensation of viral RNA potentially dysregulates viral RNA replication, localization, and packaging, our data provide a molecular explanation for how G3BP antagonizes some viral infections and why G3BP is a target for inactivation by viral proteins (*13*, *14*). However, viruses could also hijack this function to promote viral translation and/or RO assembly, as observed during norovirus infection (*39*). While our data suggest that G3BP1 proteins do not generally alter innate immune signaling pathways (Figs. 1 to 3), it remains possible that G3BP-mediated viral RNA condensation could alter the activation of these pathways via yet-to-be determined mechanisms during specific viral infections. Further work will aim to understand the consequences of G3BP-mediated viral RNA condensation and how viruses have evolved to counteract and/or hijack this intrinsic antiviral mechanism.

## MATERIALS AND METHODS

### Cell culture

The generation of RL-KO, PKR-KO, and RNase L/PKR-KO A549 cell lines is described in (*25*). The generation of WT and RL-KO cells expressing ACE2 is described in (*42*). G3BP1/2-KO A549 cells (WT and RL-KO) were generated using single gRNAs and methods described in (*26*). Cells were maintained at 5% CO_2_ and 37°C in Dulbecco’s modified Eagle’s medium supplemented with fetal bovine serum (10%, v/v) and penicillin/streptomycin (1%, v/v). Cells (12-well format; 70% confluent) were transfected with poly(I:C) high molecular weight (HMW) (InvivoGen: tlrl-pic) using 3 μl of Lipofectamine 2000 (Thermo Fisher Scientific) per 500 ng of poly(I:C).

### Viral infections

WNV (NY 99) and ZIKV (PB 81) stocks were generated in Vero cells, and the titers were determined via plaque assay on Vero cells. A549 cells (12-well format) were incubated with 0.5 ml of fetal bovine serum–free medium containing virus for 2 hours with periodic rocking. Normal growth medium was added after 2 hours, and cells were fixed 24 hours after infection in 4% paraformaldehyde. SARS-CoV-2 (WA 2020 strain) stocks generated in Vero cells were used at a multiplicity of infection (MOI) of 5 plaque-forming units (PFU)/ml to infect A549 cells (six-well format). Cells were incubated with the virus for an hour with periodic rocking. Virus inoculum was removed after 1 hour by washing with phosphate-buffered saline. Normal growth media supplemented with tested drugs were added to infected cells before incubation. Cells were fixed at selected time points in 4% paraformaldehyde. All viral infections were performed in a BSL3 facility at either Colorado State University or The Herbert Wertheim UF Scripps Institute for Biomedical Innovation & Technology under BSL3 biosafety protocols.

### Plasmids

Plasmids encoding G3BP1 and G3BP1-F124W were made by generating PCR amplicons with compatible ends (15–base pair homology) with pLenti-EF1-Blast^R^ vector. The PCR amplicons were then inserted between the xho 1 and xba 1 sites of pLenti-EF1-Blast^R^.

### Generation of cell lines expressing exogenous G3BP1

Lentiviruses were made as described in (*26*). Briefly, human embryonic kidney–293T cells (T-25 flask; 80% confluent) were cotransfected with 2.4 μg of lentiviral transfer plasmid (pLenti-EF1-Blast^R^), 0.8 μg of pVSV-G, 0.8 μg of pRSV-Rev, and 1.4 μg of pMDLg-pRRE using 20 μl of Lipofectamine 2000. Medium was collected at 48 hours after transfection and filter-sterilized with a 0.45-μm filter. Cells were transduced as described in (*25*).

### Single-molecule fluorescence in situ hybridization

smFISH was performed as described in (*25*). Glyceraldehyde-3-phosphate dehydrogenase (GAPDH) probes labeled with Quasar 570 Dye were purchased from Stellaris (GAPDH: SMF-2026-1). Custom smFISH probes for SARS-CoV-2, WNV, and ZIKV are listed in data S1.

### Microscopy and image analysis

Cover slips were mounted on slides with VECTASHIELD Antifade Mounting Medium with 4′,6-diamidino-2-phenylindole (DAPI) (Vector Laboratories, H-1200). A Nikon Eclipse Ti2 equipped with a Yokogawa CSU-W1 Spinning Disk Confocal, 100×, 1.45 numerical aperture oil objective was used for imaging with a Nikon elements software. Ten *z* planes at 200-nm steps were captured. Images were deconvoluted in Nikon elements and then processed using ImageJ with FIJI plugin. *Z* planes were stacked, and minimum and maximum display values were set in ImageJ for each channel to properly view fluorescence. The plot profile function in ImageJ was used to generate line intensity graphs. Independent replicates were performed to confirm results.

### Antibodies

IRF-3 (D6I4C) XP rabbit monoclonal antibody (Cell Signaling Technology, 11904) was used at 1:1000 for WB and IF analyses. Rabbit anti–phospho-IRF3 (Ser^396^) (D601M) (Cell Signaling Technology, 29047S) was used at WB and IF analyses. Mouse monoclonal anti-G3BP antibody (Abcam, ab56574) was used at 1:1000 for WB and IF analyses. Rabbit anti-EIF2S1 (phospho-S51–eIF2α) monoclonal antibody (Abcam, ab32157) was used at 1:1000 for WB and IF analyses. Rabbit anti-eIF2α (Cell Signaling Technology, CST9722S) was used at 1:1000 for immunoblot (IB) analysis. Rabbit anti-GAPDH (Cell Signaling Technology, 2118 L) was used at 1:2000 for IB analysis. Rabbit anti-PKR (Cell Signaling Technology, 12297S) was used at 1:1000 for IB analysis. Rabbit polyclonal anti-PABP antibody (Abcam, ab21060) was used at 1:1000 for IF analysis. Goat anti-mouse immunoglobulin G (IgG) H&L fluorescein isothiocyanate (FITC) (Abcam, ab97022) was used at 1:1000 for IF. Goat anti-rabbit IgG H&L Alexa Fluor 647 (Abcam, ab150079) was used at 1:1000 for IF. Anti-rabbit IgG, horseradish peroxidase (HRP)–linked antibody (Cell Signaling Technology, 7074S) was used at 1:3000 for WB analysis. Anti-mouse IgG, HRP-linked antibody (Cell Signaling Technology, 7076S) was used at 1:3000 for WB analysis. Antipuromycin (MilliporeSigma, MABE343) was used at 1:1000 for IF and WB analyses. Rabbit MDA-5 (Cell Signaling Technology, 5321T), rabbit MAVS (Cell Signaling Technology, 3993T), rabbit MAVS (Bethyl Laboratories, catalog no. A300-782A), and rabbit RIG-I (LSBio, LS-C331000) were used at 1:1000 for IF analyses. Goat anti-rabbit IgG H&L (Alexa Fluor 647) (Abcam, ab150079) and goat anti-mouse IgG H&L (FITC) (Abcam, ab97022) were used at 1:1000 with 2-hour incubation.

### qRT-PCR assays

qRT-PCR is described in (*25*). RNA was purified from cells using TRIzol reagent (Thermo Fisher Scientific, 15596026) and treated with deoxyribonuclease I. RNA was precipitated via sodium acetate ethanol precipitation, and 500 ng of total RNA was converted to cDNA using SuperScript III and poly(dT) 20 primer. cDNA was generated from RNA collected from at least three independent experiments at the same time. The cDNA was then diluted 1:5, and qPCR was performed using 1 μl of diluted cDNA and gene-specific primers for IFNB (*25*). The qPCR reactions were performed in duplicate to confirm technical efficacy. The technical replicates were then averaged to generate the *C*_t_ value of each biological replicate.

### Enzyme-linked immunosorbent assay

Twelve hours after lipofection of poly(I:C), 50 μl of medium was assayed using IFNB human enzyme-linked immunosorbent assay (ELISA) kit (Thermo Fisher Scientific, 414101) via the manufacturer’s instructions.

### Statistical analyses

*P* values were derived via Student’s *t* test. *P < 0.05, **P < 0.01, and ***P < 0.001. Quantification of data is in data S1.
