# Supplementary material for: G3BP1-dependent condensation of translationally inactive viral RNAs antagonizes infection
Source: Sci Adv. 2024 Jan 31;10(5):eadk8152. doi: 10.1126/sciadv.adk8152 (PMC10830107; doi:10.1126/sciadv.adk8152)
Supplement: Supplementary file 1 — Figs. S1 to S12 Legend for data file S1 [file sciadv.adk8152_sm.pdf]

Science Advances
AAAS

Supplementary Materials for

G3BP1-dependent condensation of translationally inactive viral RNAs antagonizes infection

James M. Burke *et al.*

Corresponding author: James M. Burke, james.burke@ufl.edu; Roy Parker, roy.parker@colorado.edu

Sci. Adv. **10**, eadk8152 (2024)
DOI: 10.1126/sciadv.adk8152

The PDF file includes:

Figs. S1 to S12
Legend for data file S1

Other Supplementary Material for this manuscript includes the following:

Data file S1

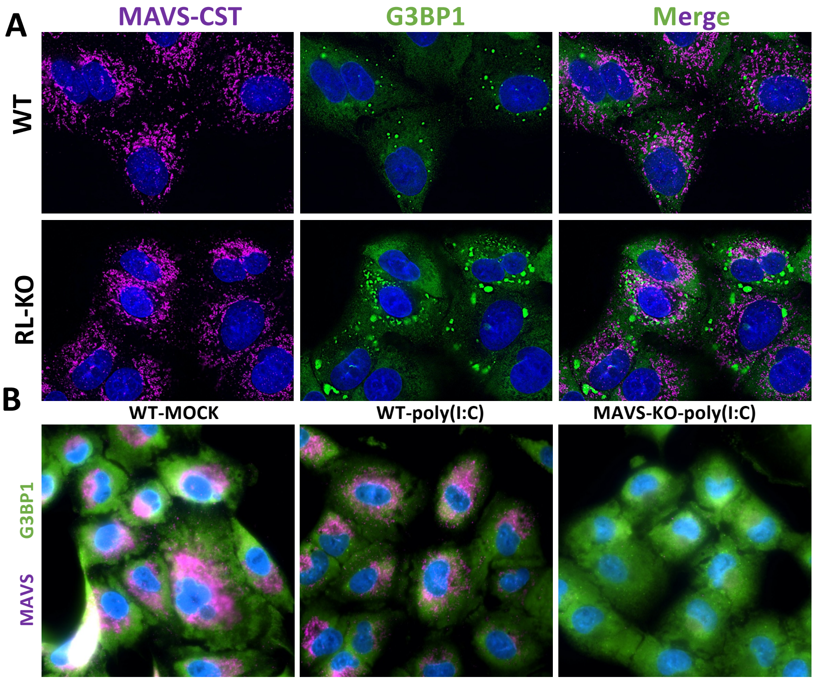

Fig. S1.
(A) Immunofluorescence assay for G3BP1 and MAVS antibody (cell signaling technologies). (B) Immunofluorescence assay for G3BP1 and MAVS (Bethyl Laboratories) in parental and MAVS-null A549 cells.

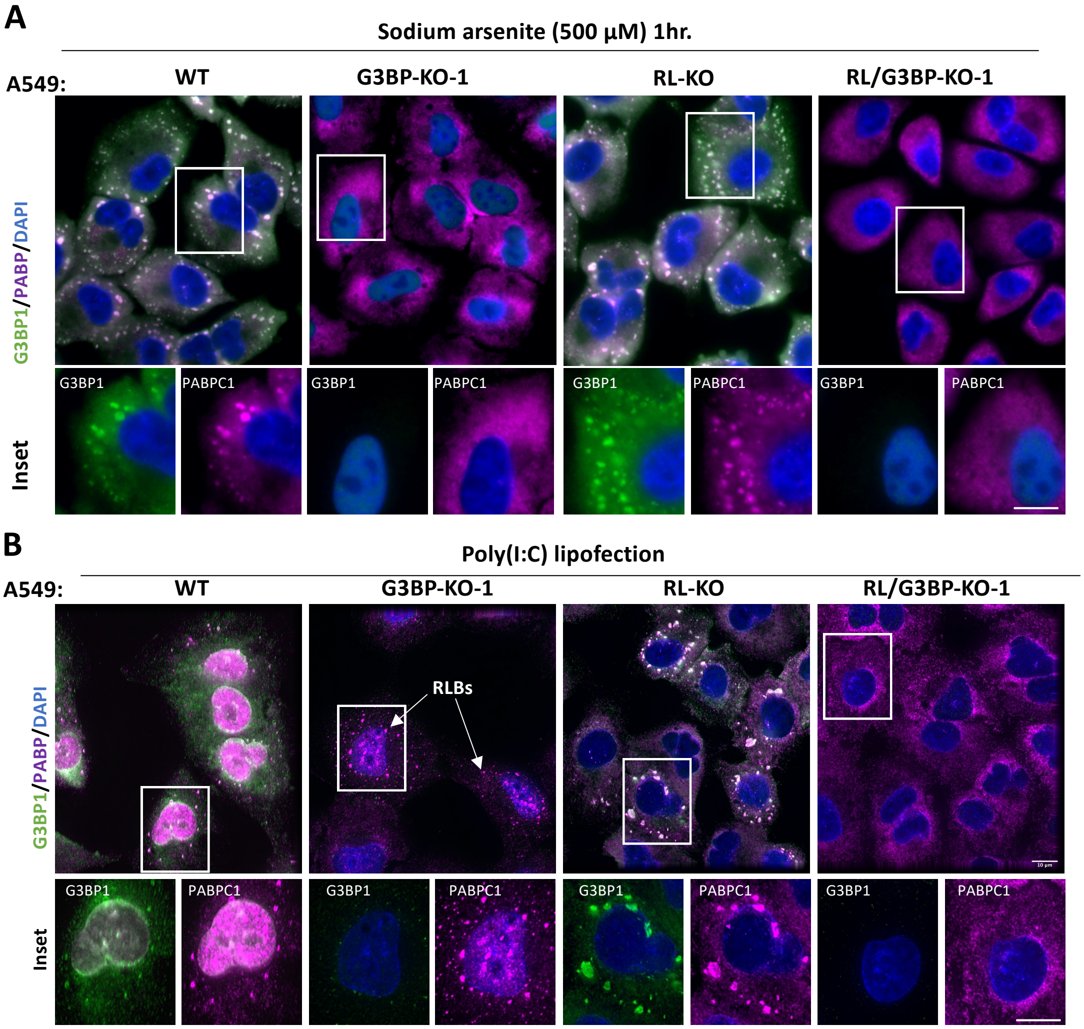

Fig. S2.
(A) IF for stress granule markers, G3BP1 and PABPC1, in indicated cell lines 1-hour post-treatment with sodium arsenite. (B) Similar to (A) but following six hours post-lipofection of poly(I:C).

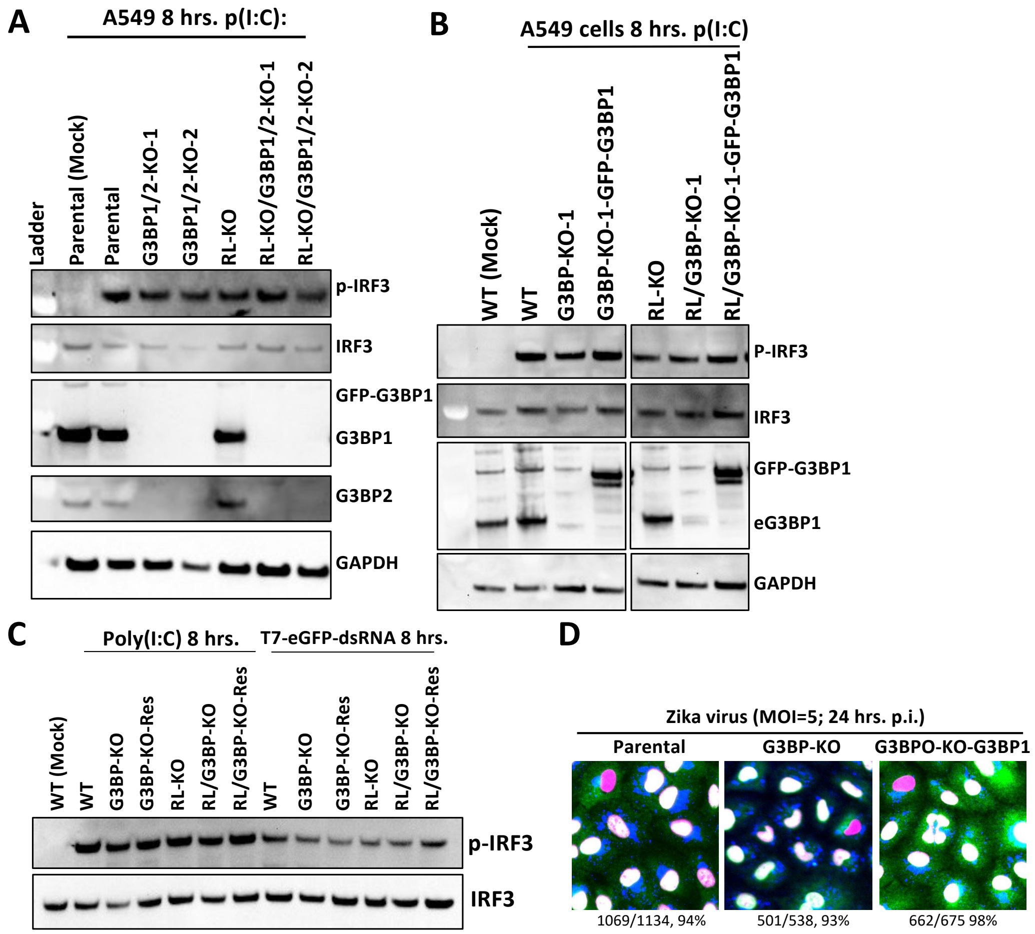

Fig. S3.

(A,B) Immunoblot assay for phosphorylation of IRF3 (p-IRF3) and G3BP proteins in indicated cell lines 8 hours post-lipofection of poly(I:C). (C) Immunoblot assay for phosphorylation of IRF3 (p-IRF3) in indicated cell lines 8 hours post-lipofection with T7-transcribed dsRNA. (D) Immunofluorescence assay for IRF3 in indicated cell lines 24 hours post-infeciton with Zika Virus (MOI=10).

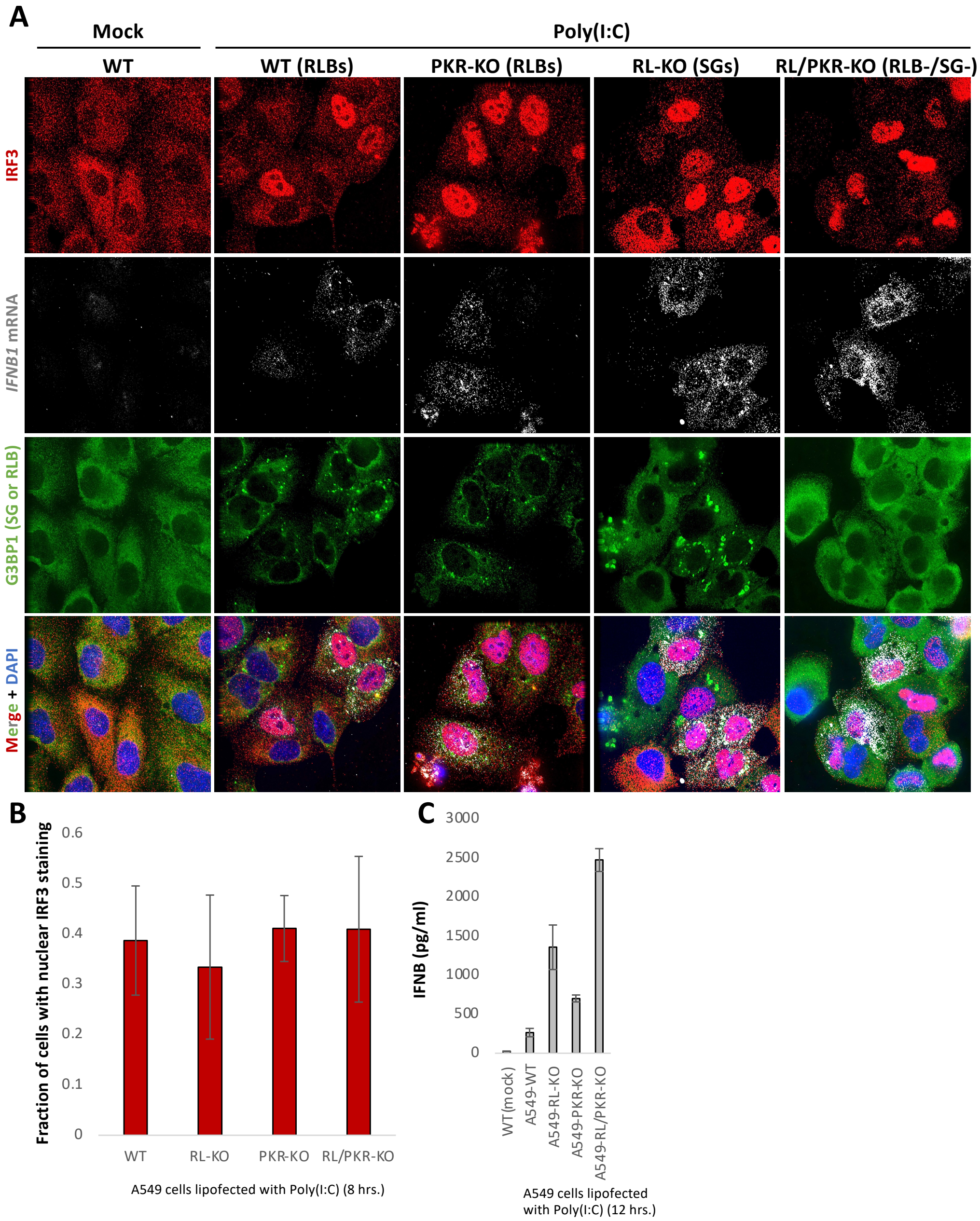

Fig. S4.
(A) Immunofluorescence assay for G3BP1 and IRF3 and smFISH for *IFNB1* mRNA in indicated cell lines 8 hours post-lipofection of poly(I:C). WT and PKR-KO cells generate RNase L-dependent bodies that stain for G3BP1. RL-KO cells generate G3BP-positive stress granules. RNase L/PKR double knockout cells do not generate G3BP1 granules (neither RLB nor SGs). IRF3 translocates to the nucleus equally in all cell lines. Most cells with nuclear IRF3 induce *IFNB1* mRNA. (B) Average +/- S.D. from four independent replicates of the percent of cells from indicated cell lines displaying nuclear IRF3 at 8 hours post-lipofection with poly(I:C) as represented in (A). Cells with IRF3 intensity equal to or higher than the cytoplasm were counted at having nuclear IRF3. (C) ELISA for IFNB in the supernatant of indicated cell lines 12 hours post-lipofection of poly(I:C).

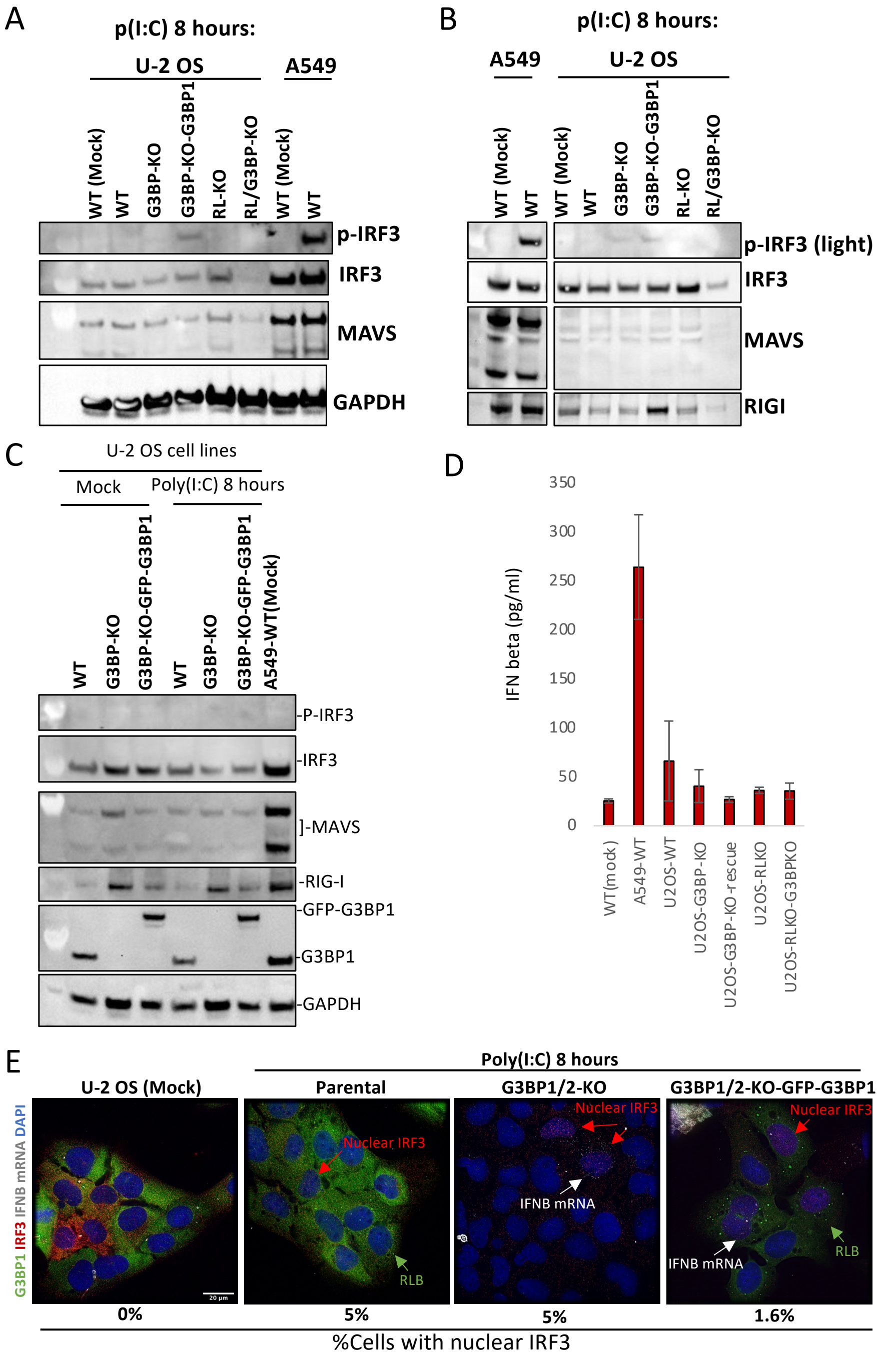

Fig. S5.
(A-C) Immunoblots for indicated proteins in either U-2 OS or A549 cell lines. (D) ELISA for IFNB1 protein secretion in indicated cell lines. Limit of detection 50 pg/ml. Bars represent the average +/- standard error from at least three independent experiments. (E) IF for G3BP1 and IRF3 and smFISH for IFNB mRNA in parental, G3BP1/2-KO, and G3BP1/2-KO-GFP-G3BP1 U-2 OS cell lines 8 hours post-lipofection with poly(I:C). Below the panels, the percentage of cells that display nuclear IRF3 stated. Greater than 300 cells from each cell line was analyzed from two independent replicates.

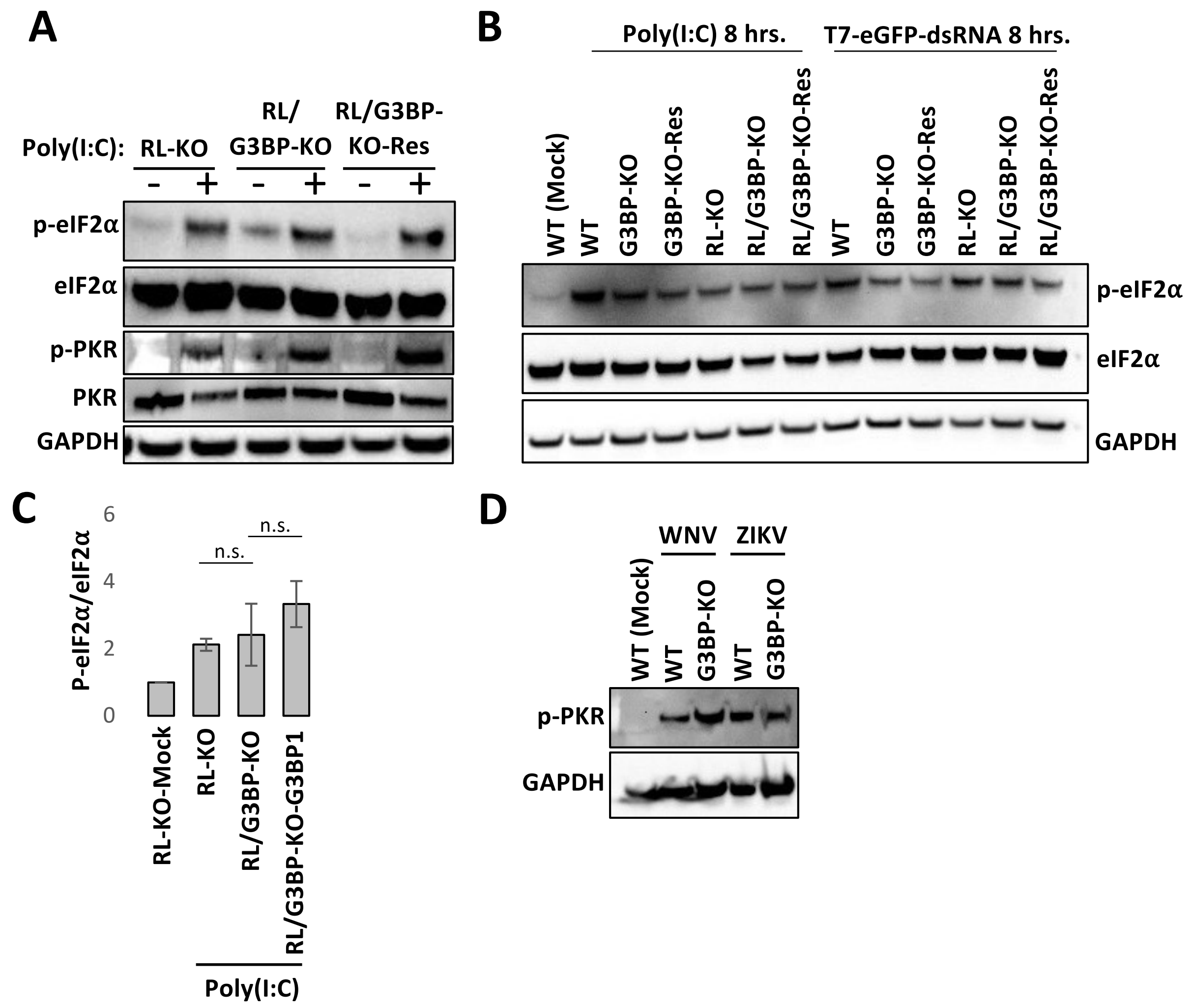

Fig. S6.
(A) Western blot analysis for phosphorylation of eIF2α (p-eIF2α) and PKR (p-PKR) eight hours post-lipofection of poly(I:C). (B) Similar to (A) but cells were lipofected with T7-generated dsRNA. (C) Mean +/- S.D. p-eIF2α/eIF2α ratio in indicated cell lines eight hours post-lipofection of poly(I:C) from three independent experiments. (D) Western blot analysis for phosphorylation PKR (p-PKR) in WT or G3BP1/2-KO cells 24 hours post-infection with either WNV or ZIKV.

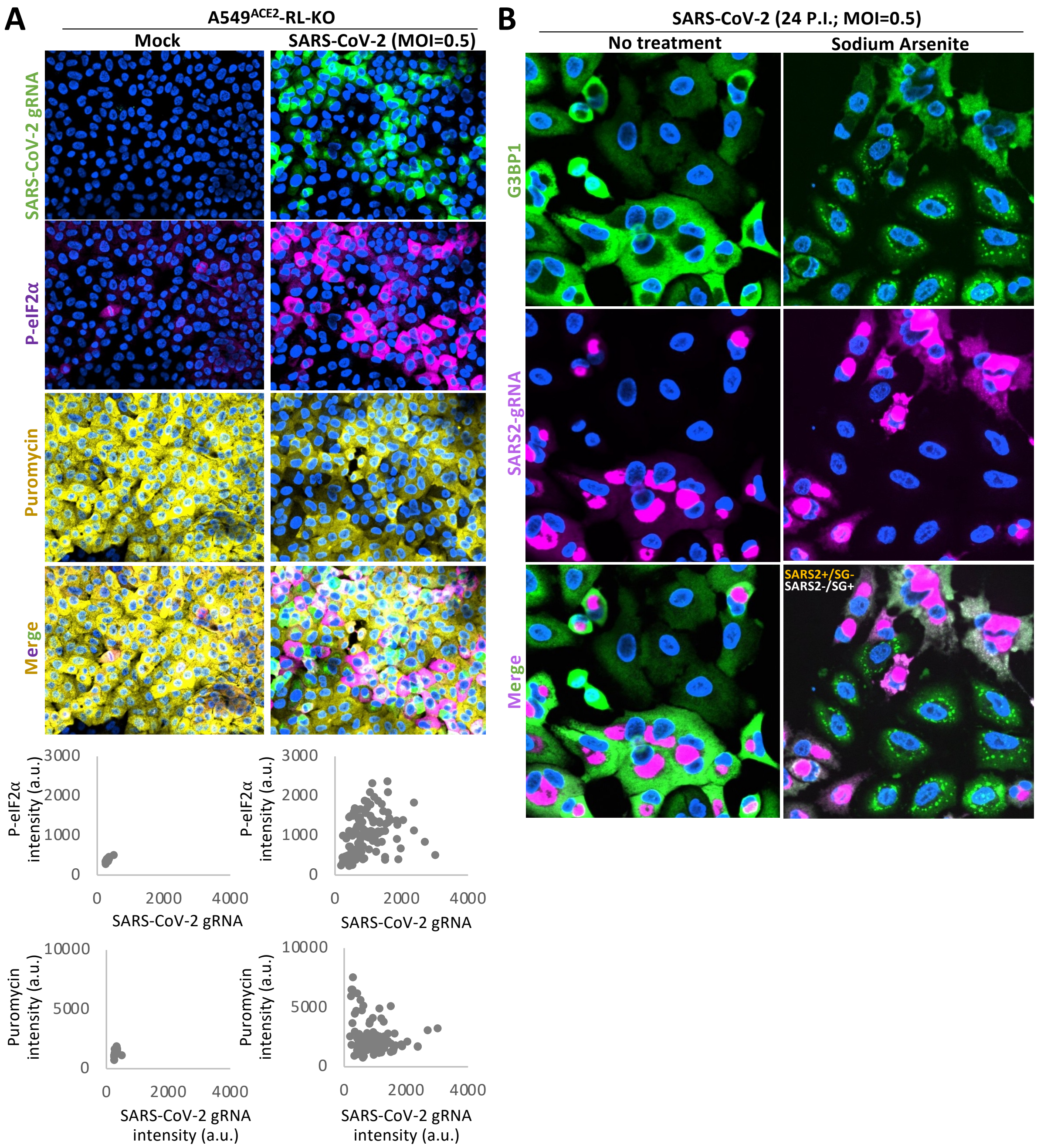

Fig. S7.

(A) Immunofluorescence assay for puromycin p-eIF2α and smFISH for SARS-CoV-2 ORF1a mRNA. (B) Quantification of mean intensity from individual cells represented in (A). (C) Immunofluorescence assay for puromycin G3BP1and smFISH for SARS-CoV-2 ORF1a. Twenty-three hours post-infection with SARS-CoV-2 cells were treated with 500 μM sodium arsenite for 1 hour. White arrows indicate non-infected cells (lack SARS-CoV-2 gRNA). Yellow arrows indicate SARS-CoV-2 infected cells (contain SARS-CoV-2 gRNA). Non-infected cells (white arrows) generate stress granules in response to sodium arsenite treatment, whereas infected cells (yellow arrows) do not.

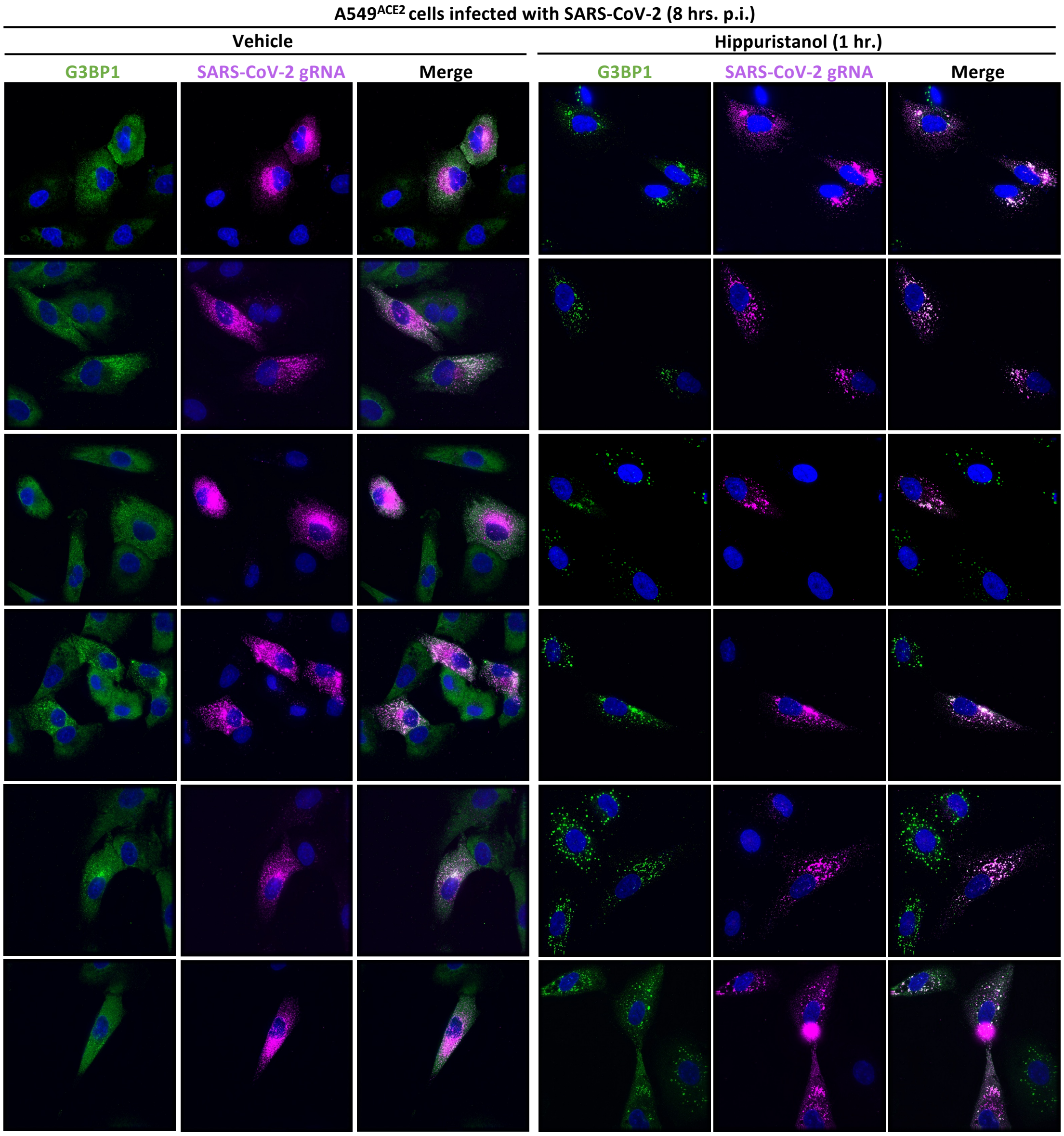

Fig. S8.
smFISH for SARS-CoV-2 ORF1a RNA and IF for G3BP1 in A549^{ACE2} cells infected with SARS-CoV-2 for eight hours and then treated with either vehicle (DMSO) or hippuristanol for 1 hour.

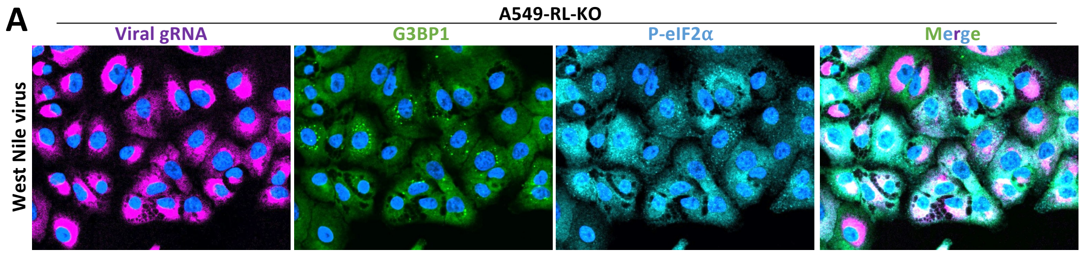

Fig. S9.
(A) Immunofluorescence for G3BP1 and p-eIF2α and smFISH for the 5'-end of viral RNAs in A549 RNase L-KO cells twenty-four hours post-infection with West Nile virus (WNV) (MOI=10).

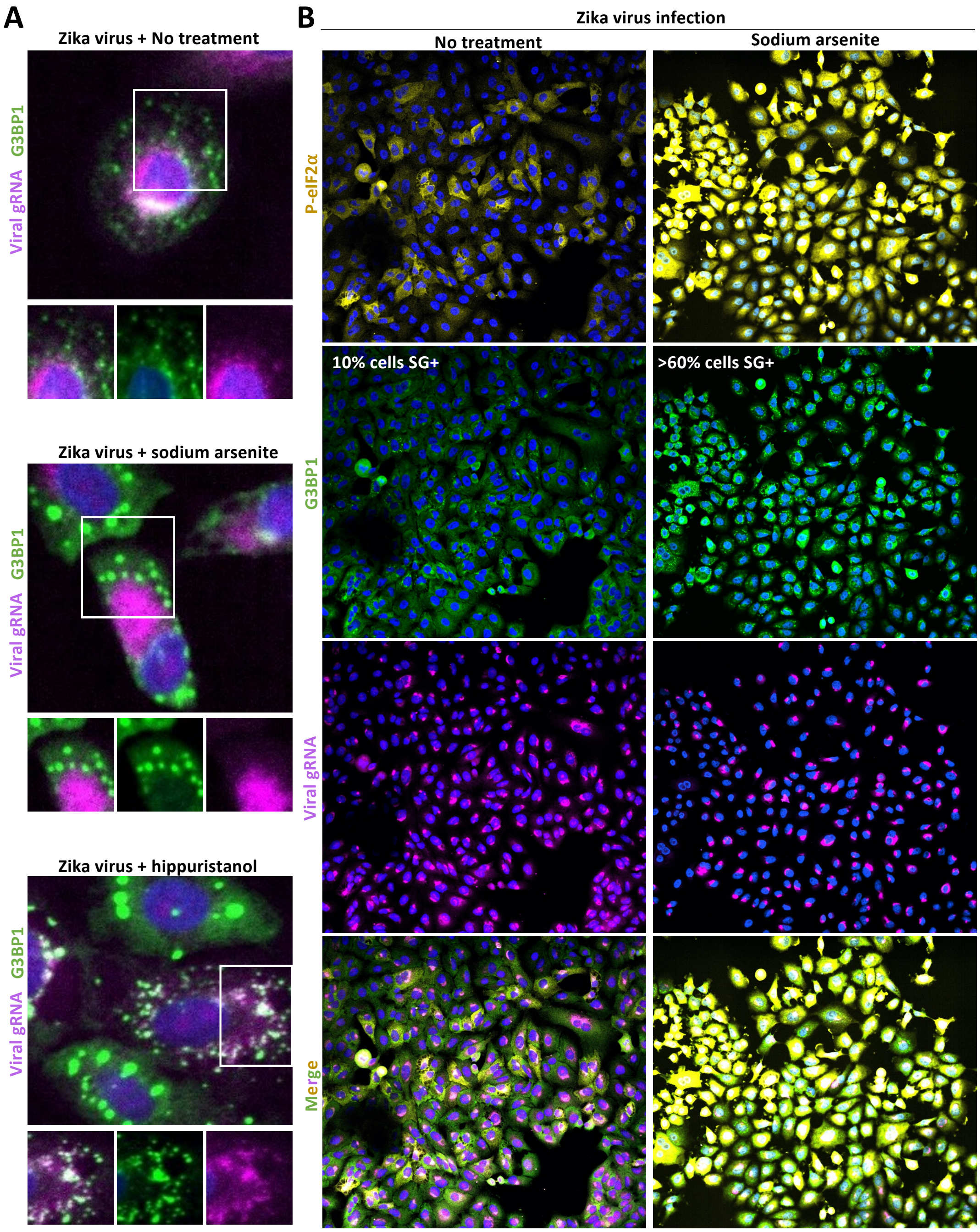

Fig. S10.
(A) Immunofluorescence assay for G3BP1 and smFISH for ZIKA virus RNA in cells treated for one hour with 500 μM sodium arsenite, 1μM hippuristanol, or 50nM of pateamine A. (B) Immunofluorescence assay for p-eIF2α and G3BP1 and smFISH for ZIKA virus genomic RNA (5'-end).

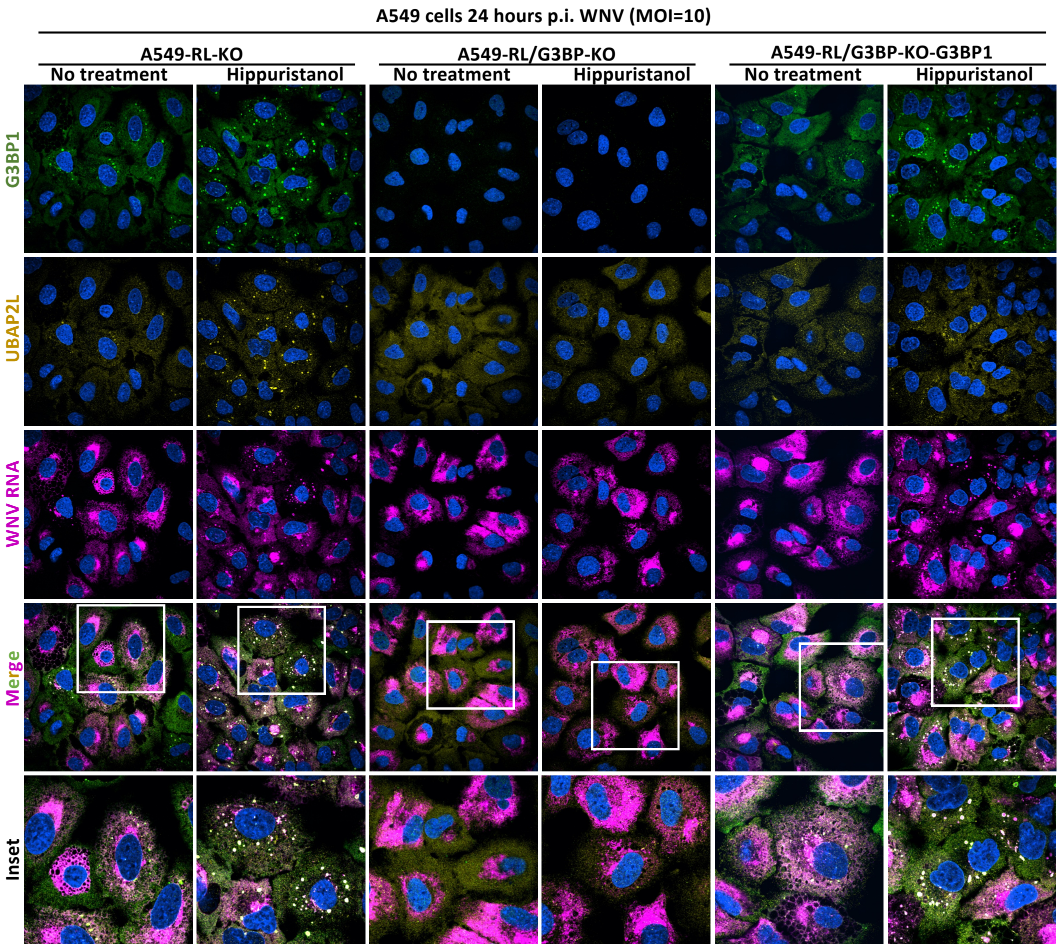

Fig. S11.
Immunofluorescence assay for G3BP1 and UBAP2L and smFISH for WNV RNA in RNase L-KO, RNase L/G3BP-KO, and RNase L/G3BP-KO A549 cell lines 24 hours post-infection with WNV (MOI=10). Cells were treated with or without 1μm hippuristanol 22 hours p.i.

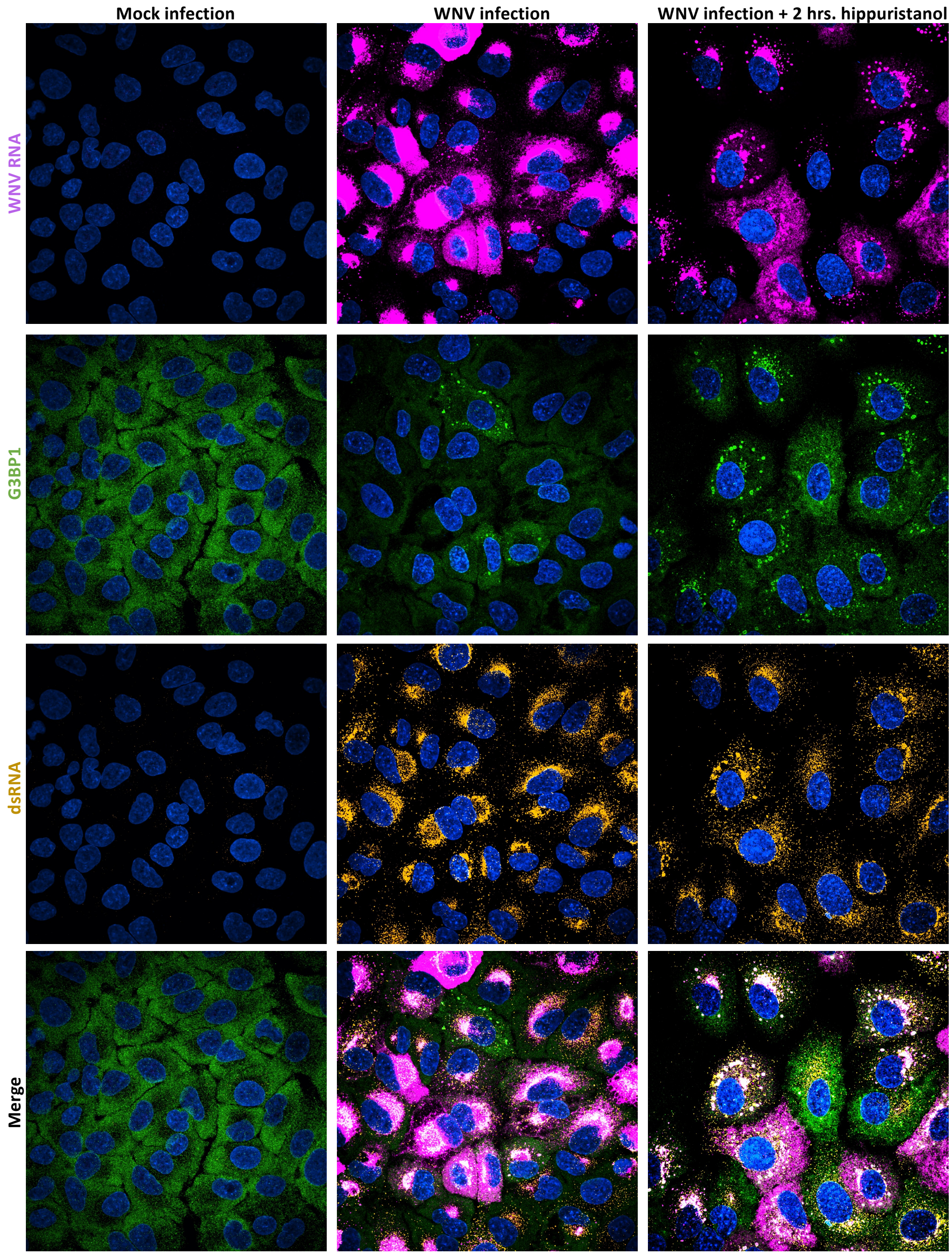

Fig. S12.

Immunofluorescence assay for G3BP1 and dsRNA and smFISH for WNV RNA in RNase L-KO A549 cell lines 24 hours post-infection with WNV (MOI=10). Cells were treated with or without 1μm hippuristanol at 22 hours p.i.

Data S1.

Spreadsheets of microscopy line traces, intensity measurements, quantification, statistical analyses, and custom smFISH probe sets.
